# Adaptive from Innate: Human IFN-γ^+^CD4^+^ T Cells Can Arise Directly from CXCL8-Producing Recent Thymic Emigrants in Babies and Adults

**DOI:** 10.4049/jimmunol.1700551

**Published:** 2017-07-28

**Authors:** Abhishek Das, Kevin Rouault-Pierre, Shraddha Kamdar, Iria Gomez-Tourino, Kristie Wood, Ian Donaldson, Charles A. Mein, Dominique Bonnet, Adrian C. Hayday, Deena L. Gibbons

**Affiliations:** * Peter Gorer Department of Immunobiology, King’s College London, London SE1 9RT, United Kingdom;; † The Francis Crick Institute, London NW1 1AT, United Kingdom;; ‡ National Institute for Health Research Biomedical Research Centre Genomics Research Platform, Guy’s Hospital, London SE1 9RT, United Kingdom; and; § Genome Centre, Barts and the London School of Medicine and Dentistry, John Vane Science Centre, London EC1M 6BQ, United Kingdom

## Abstract

We recently demonstrated that the major effector function of neonatal CD4^+^ T cells is to produce CXCL8, a prototypic cytokine of innate immune cells. In this article, we show that CXCL8 expression, prior to proliferation, is common in newly arising T cells (so-called “recent thymic emigrants”) in adults, as well as in babies. This effector potential is acquired in the human thymus, prior to TCR signaling, but rather than describing end-stage differentiation, such cells, whether isolated from neonates or adults, can further differentiate into IFN-γ–producing CD4^+^ T cells. Thus, the temporal transition of host defense from innate to adaptive immunity is unexpectedly mirrored at the cellular level by the capacity of human innate-like CXCL8-producing CD4^+^ T cells to transition directly into Th1 cells.

## Introduction

The discovery that neonatal T cells are extremely poor producers of IFN-γ has fueled the hypothesis that the neonatal immune system is intrinsically anti-inflammatory, perhaps to perturb overexuberant responses to self-antigens and nonpathogenic commensals. However, this presumably must accommodate mechanisms to protect against foreign pathogens (1). In this regard, we found, surprisingly, that human CD4^+^ T cells from preterm and term neonates harbor proinflammatory capacity in the form of CXCL8 production, an antimicrobial chemokine with a potent capacity to recruit neutrophils (2–4).

A key question raised by this finding was whether CXCL8-producing CD4^+^ T cells are inherently different from adult effector T cells, consistent with the reported discontiguity of fetal and adult hematopoiesis (5), or whether newly arising CD4^+^ T cells in adult humans can likewise produce CXCL8. Were this to be the case, do such cells comprise a hitherto overlooked “Th8” subset, or are they developmental intermediates in the differentiation of other human T cell lineages? For example, IFN-γ/IL-13–producing murine dendritic epidermal γδ T cells arise from progenitors with the potential to produce IL-17 (6). Moreover, it was reported that IL-17–producing αβ T cells are not necessarily a differentiation end point but may convert to IFN-γ production (7). These observations are particularly germane, given that mice lack CXCL8, with some of its functions including neutrophil recruitment attributed to IL-17 (8, 9).

To address these key questions, we have tracked the development of human CD4^+^ effector function from the thymus to the periphery. We show that CXCL8 expression is imprinted early during thymic development, prior to TCR signaling, that it is retained in T lineage–committed cells, and that it is subsequently enriched in recent thymic emigrants (RTEs) in adults, as well as in children. Furthermore, we show that CXCL8^+^ CD4^+^ T cells from neonates or adults can serve as direct precursors of human IFN-γ–producing CD4^+^ Th1 cells. Thus, the most naive peripheral T cells in adults, as well as in neonates, have the potential for CXCL8 production, a fact that may have been overlooked because of reduced thymic output in adults and the cells’ rapid conversion to Th1 cells. These data provide novel insights into human T cell ontogeny, illustrating how conventional adaptive lymphocyte immunity can arise from innate-like cells, rather than from functionless T cell progenitors (Th0 cells), as has been considered the norm.

## Materials and Methods

### Human samples

Normal human umbilical cord blood (CB) samples were obtained from the Royal London Hospital under ethical approval (HRECO6/Q0604/110) and from the Anthony Nolan Research Biobank (15/EM/0045). Thymus tissue was obtained from the Evelina Children’s Hospital (09/H0504/39). Blood from children was obtained in collaboration with Dr. C. Furness and Prof. M. Greaves at the Institute for Cancer Research using United Kingdom Childhood Cancer Study stored samples from patients with nephroblastoma or neuroblastoma (CCR2285); adult blood was obtained from healthy volunteers (07/H0803/237). T cell acute lymphocytic leukemia (T-ALL) samples were obtained from the Bloodwise Childhood Leukaemia Cell Bank (16/SW/0219). Mononuclear cells were isolated from blood by centrifugation using Ficoll-Paque PLUS (GE Healthcare Life Sciences). For CB, mononuclear cells were enriched in CD34^+^ cells (STEMCELL Technologies), and the CD34^−^ fraction (on occasion from pooled donors) was used for experiments.

### Immunodeficient mice

NOD-SCID IL-2Rγ^null^ (NSG) mice were a kind gift from Dr. Leonard Shultz (The Jackson Laboratory, Bar Harbor, ME). Twenty-four hours before transplantation, mice were sublethally irradiated (375 cGy). CD34^+^ cells were transplanted into 8–12-wk-old NSG mice using intrabone marrow injection, and blood samples were collected from the tail at different time points. All animal work was undertaken in accordance with UK Home Office guidelines.

### Flow cytometry and cell sorting

Individual cell subsets were sorted (FACSAria II; Becton Dickinson) from thymocytes, CB, or PBMCs using BioLegend Abs: anti-CD4 Pacific Blue (OKT4), anti-CD8α allophycocyanin Cy7 (HIT8A), anti-CD34 Alexa Fluor 647 (581), anti-CD7 PE Cy7 (CD7-6B7), anti-CD3 allophycocyanin Cy7 (HIT3a), anti-CD31 Alexa Fluor 647 (WM59), and anti-CD45RA PECy7 (HI100; BD Pharmingen).

Flow cytometry analysis was performed using a BD FACSCanto II. Cells were stained with LIVE/DEAD fixable aqua stain (Thermo Fisher) prior to staining with the Abs above and anti-TCR γδ PE Cy7 (B1), anti-CD5 PerCP Cy5.5 (L17F12), or anti-CD1a PE (HI149; all from BioLegend). For intracellular staining, cells were fixed (CellFIX; BD), permeabilized (Perm/Wash Buffer; BioLegend), and stained with anti-CXCL8 FITC (E8N1), anti–IFN-γ PerCP Cy5.5 (4S.B3), anti–IL-2 PerCP Cy5.5 (MQ1-17H12), or anti–IFN-γ PE (B27).

For TCR excision circle (TREC) PCR, a modified intracellular cytokine staining protocol was used to maximize DNA quality postexposure to fixative. Cells were sorted after surface staining in PBS with anti-CD4 Pacific Blue, anti-CD3 allophycocyanin Cy7, anti-CD45RA PE/Cy7, and anti-CD31 Alexa Fluor 647 (BioLegend), followed by fixation in 1% paraformaldehyde (20 min) and intracellular staining for anti-CXCL8 FITC and anti–IL-2 PerCP Cy5.5 (BioLegend) in 0.1% saponin (25 min). Two additional washes in cold PBS were performed prior to acquisition.

### Cell stimulation

Total or individually sorted cells were stimulated with PMA (10 ng/ml) and ionomycin (1 μg/ml; both from Sigma) or anti-CD3+CD28 Dynabeads (1:1 ratio with cells; Thermo Fisher) in complete medium (RPMI 1640 medium containing 100 U penicillin and 100 μg/ml streptomycin [Invitrogen] and 10% FCS [STEMCELL Technologies]). For intracellular FACS, Brefeldin A (BFA; 20 μg/ml; Sigma) was added. In assays in which prolonged stimulation was required, IL-7 (10 ng/ml; PeproTech) was added. For cell tracer experiments, CD4^+^ T cells were stained with a CellTrace Violet Cell Proliferation Kit (Thermo Fisher). A total of 0.2 × 10^6^ cells were then resuspended in CM containing IL-7 (10 ng/ml), with or without anti-CD3+CD28 beads. After 5 d, cells were restimulated with PMA plus ionomycin (PI) prior to cytokine analysis.

### Real-time PCR for TRECs

To preserve DNA quality within fixed cells, intracellular cytokine staining was modified as described above. DNA was extracted from individual sorted cell subsets using the DNeasy Blood and Tissue Kit (QIAGEN), using a modified protocol. In brief, 10 μl of proteinase K was added to cells suspended in 200 μl of PBS, followed by Buffer AL. Cells were incubated at 56°C for 12 min and then for 1 h at 80°C, with agitation prior to the addition of ethanol. The remaining steps were carried out as per the kit manufacturer’s protocol. Signal joint TREC numbers were quantified with a real-time PCR-based assay, as described previously (10). DNA concentration was assessed using a NanoDrop Spectrophotometer, and the ratio of absorbance at 260 and 280 nm was used to assess purity. An absorbance ratio > 1.8 was achieved in the majority of cases; however, in all cases, quality was sufficient for quantitative PCR (qPCR).

### FluoroSpot

IFN-γ/CXCL8 production was assessed in sorted CD4^+^ T cells using the IFN-γ/CXCL8 FluoroSpot Kit (Mabtech). Stimulation conditions were overnight (20 h): PI or anti-CD3+CD28 beads (1:1 ratio with cells) and 72 h: anti-CD3+CD28 beads, followed by PI for the last 20 h of stimulation. Duplicate wells were set up for each stimulation condition. IL-7 (10 ng/ml) was added to all wells. Enumeration of fluorescent spots was automated using a Fluorospot plate reader (BIOREADER 6000-F beta; Bio-Sys).

### T cell cloning from FluoroSpot plates

Negatively selected CD4^+^ T cells from CB (CD4^+^ T Cell Isolation Kit, human; Miltenyi Biotec) were incubated for 2 h with PI. They were then stained and single cell sorted (gated on 7-aminoactinomycin D negative, CD3^+^CD4^+^CD31^+^CD45RA^+^ T cells) directly into a 96-well CXCL8/IFN-γ FluoroSpot plate containing 100 μl per well of X-VIVO 15 medium (Lonza) and 10% human AB serum. Following a 20-h incubation to allow cytokine capture on the membrane, individual cells were removed with a pipette from the FluoroSpot plate and transferred directly into the corresponding well of a 96-well round-bottom plate for cloning. An additional 50 μl of X-VIVO medium containing 10% human AB serum was used to wash each FluoroSpot well to increase the probability of cell recovery and transferred into the 96-well round-bottom plate. The FluoroSpot plate, now with all cells removed, was washed, and the assay was conducted according to the manufacturer’s instructions.

To clone transferred single cells, irradiated APCs (1:100,000) and PHA (4 μg/ml; Thermo Fisher) were added to make the final volume in the well 200 μl. Additional irradiated APCs were added at days 10, 25, and 38. T cell growth factor (5%; Helvetica Health Care) was added to wells after day 3. At days 35 and 53, clones were isolated, surface stained, and intracellularly stained for anti–IL-17A allophycocyanin (BL168), anti–IL-4 PE (MP4-25D2), anti–IL-2 PE Cy7 (MQ1-17H12), anti–IFN-γ PerCP Cy5.5 (4S.B3), and anti-CXCL8 FITC (BioLegend). At the same time points, cells were sorted for TCR sequencing to validate clonality (see below).

### Validation of single-cell FluoroSpot assay

Because this was the first description, to our knowledge, of single-cell sorting onto a FluoroSpot plate and acquisition thereafter, the following validation steps were implemented. First, cell counting was automated by the Bio-Sys BIOREADER and quantitated as 0, 1, or >1 spots. A total of 544 wells were analyzed. Any well with 0 or >1 spot was discarded; only wells counted as “1” were analyzed further. Second, to identify a single CXCL8-producing cell, all wells with a single red spot (CXCL8 Cy3^+^; red) were included. Any well with a green spot (IFN-γ FITC^+^; green) or a yellow spot (CXCL8 Cy3^+^ + IFN-γ FITC^+^) was discarded. Third, all wells with a single red spot were scrutinized for atypical morphology (most likely representing debris) and, if present, were discarded. Finally, all wells with a single red spot (CXCL8 Cy3^+^) meeting the above criteria were reimaged on the AID Bioreader as further validation. Thus, 29 of 544 wells (5.3%) were deemed to have a single Cy3^+^ signal.

### Single-cell RNA sequencing

‪CD4^+^ T cells from CB samples (*n* = 2) were isolated (negative selection) by cell sorting and activated for 2.5 h with PI. Single cells were captured using the C1 Single-Cell Auto Prep System (Fluidigm, San Francisco, CA) along with the SMARTer kit for cDNA generation (Clontech, Mountain View, CA). One microliter of ERCC spike-in mix (Life Technologies, Carlsbad, CA) was included (1:1000). Briefly, 1000 cells were loaded onto each array (C-Chip; INCYTO, Cheonan-si Chungnam-do, Korea). The first array yielded 43 single cells, and the second yielded 61. cDNA quantity and quality were measured using a Qubit 2.0 Fluorometer (Life Technologies) and an Agilent 2200 TapeStation (Agilent Technologies, Waldbronn, Germany). A total of 96 single cells (55 from one cord and 41 from another) were chosen for RNA sequencing, selecting cells with a range of CXCL8 expression (as determined by real-time PCR). Libraries were prepared using the Illumina Nextera XT Sample Preparation Kit (Illumina, Cambridge, U.K.). Seventy-five–base pair paired-end reads were generated using the Illumina NextSeq 500 System. Paired-end RNA sequencing reads from these samples were simultaneously aligned to the human genome (GRCh37/hg19) and ERCC92 spike-in control sequence using TopHat v2.0.4 (11) and Bowtie2 v2.0.0.beta6 (12)‬. ‬Raw sequence counts mapped to genes or ERCC spike-in controls were obtained using htseq-count (13). Total gene counts were normalized to ERCC spike-in control read counts using DESEquation (14). Differentially expressed genes were those that showed statistically significant evidence [after multiple hypothesis correction using a false discovery rate of 0.1 (15)] against the null hypothesis that their biological coefficient of variation was <50% (i.e., CV^2^ < 0.25) (16).‬‬‬‬ Data have been submitted to the Gene Expression Omnibus under accession number GSE84686 (https://www.ncbi.nlm.nih.gov/geo/).‬‬‬‬‬‬‬‬‬‬‬‬‬‬‬‬‬‬‬‬

### Single-cell TCR sequencing

Sorted single live CD4^+^ T cells were collected, and single-cell nested PCR for TCRA was performed as described previously (17). PCR products were Sanger sequenced using the HTSP-tag primer, results were referenced to the IMGT database (18), and the CDR3A sequences were compared across individual cells using KNIME 2.11.2 (19).

### TCR sequencing from whole-cell pellets

Sorted live CD4^+^ T cells were collected, snap-frozen, and stored at −80°C. Whole-cell pellets were sent to Adaptive Biotechnologies (Seattle, WA) for DNA extraction and TCR sequencing. Cells were identified as clonal based on confidence levels established by Adaptive Biotechnologies.

### Statistical analysis

A nonparametric paired Wilcoxon test was used for all paired data. Error bars on graphs represent SD around the mean.

## Results

### CD4^+^ RTEs in adults express CXCL8

**FIGURE 1.**
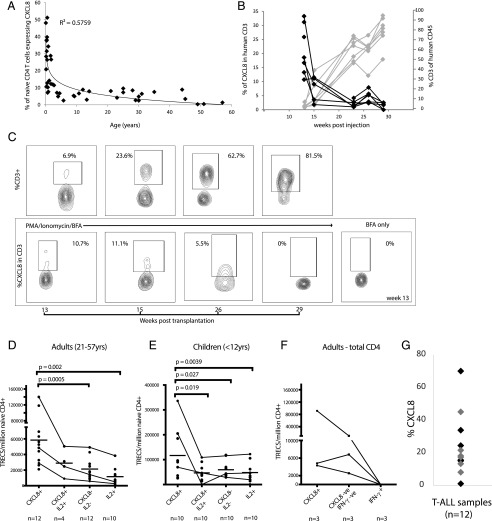
CXCL8-producing T cells decline with age in humans and in vivo in humanized mice and are enriched in RTEs. (**A**) CXCL8 production was determined in naive CD4^+^ T cells (4 h PI + BFA) obtained from 45 individuals (aged between 3 mo and 57 y). (**B**) Irradiated NSG mice were reconstituted with human CD34^+^ cells (CB derived). The graph shows reconstitution of T cells over time in individual mice (gray lines), with a reciprocal decline in T cell production of CXCL8 (black lines). (**C**) FACS plots show an example of this reciprocal change in one reconstituted NSG mouse; percentage of cells expressing CD3 within human CD45^+^ cells (upper panels) and percentage of cells expressing CXCL8 among CD3 T cells (lower panels; PI + BFA or BFA alone for 4 h). CD4^+^ T cells from adults (**D** and **F**) or children aged 1–12 y (**E**) were activated with PI (4 h), and cytokine production was determined by intracellular staining. Individual (cytokine^+^) subsets were then sorted, and TREC content was determined by qPCR. Results are shown as TREC levels per million naive CD4^+^ cells or TREC levels per million total CD4^+^ cells in (F), because very few naive CD4^+^ cells express IFN-γ. Trend lines depict the differences in TREC levels among the four sorted populations in three patients. (**G**) CXCL8 production was determined following activation in vitro with PI (4 h, in the presence of BFA) in primary T-ALL samples (*n* = 12); later stages (T-III/T-IV) are represented by gray diamonds.

CXCL8 production is the most highly represented functional potential among naive CD4^+^ T cells from neonates (2). Thus, when naive CD4^+^ T cells (as determined by CD45RA and CD31 expression) from 45 donors of different ages were examined following polyclonal activation, CXCL8 production was highest in babies and was strongly and negatively correlated with age (*R*^2^ = +0.57, *p* < 1.7 × 10^−9^). Nevertheless, CXCL8-producing T cells accounted for ∼10% of naive CD4^+^ T cells of several donors aged between 20 and 45 y (Fig. 1A).

A similar observation was made in NSG mice humanized by inoculation with CD34^+^ hematopoietic stem progenitor cells following irradiation. PI induced CXCL8 production, by up to >30% of the first-arising human T cells at ∼13–15 wk postinoculation; thereafter, CXCL8 production declined dramatically as the representation of human CD3^+^ T cells increased (Fig. 1B, 1C).

The age-related decline in the capacity of naive human T cells to produce CXCL8 mirrored the documented decline in thymic output (20). Therefore, to determine whether CXCL8 production was a feature unique to RTEs, it was measured in relation to TRECs, which are the most reliable (and clinically applied) marker of RTEs (10, 21). CD4^+^ T cells from adults aged 21–57 y were stimulated with PI for 4 h and stained for intracellular cytokine production using a protocol that preserves DNA quality. Subsets of naive CD31^+^CD45RA^+^ cells that expressed CXCL8, CXCL8 plus IL-2, IL-2 alone, or neither CXCL8 nor IL-2 were isolated by flow cytometry, and TREC levels were assessed by qPCR. CXCL8^+^ cells were highly enriched in TRECs relative to cells producing IL-2 alone (*p* = 0.002) or neither cytokine (*p* = 0.0005) and contained more TRECs than cells coproducing CXCL8 and IL-2, as shown by individual tracking lines (Fig. 1D). Likewise, CXCL8^+^CD4^+^ T cells from children (*n* = 10, aged <12 y) were highly enriched in TRECs (Fig. 1E). Thus, CXCL8 production was associated with the most immature of naive peripheral T cells in adults, as well as in children. In contrast, IFN-γ–producing CD4^+^ cells from each of three adults examined were almost completely depleted of TRECs (Fig. 1F). Interestingly, another scenario in which CXCL8 production was associated was primary T-ALL samples including those with a more mature T cell (T-III/T-IV) classification (22) (gray diamonds, *n* = 6 of 12 T-ALL samples, Fig. 1G)

### CXCL8 imprinting in thymocytes

**FIGURE 2.**
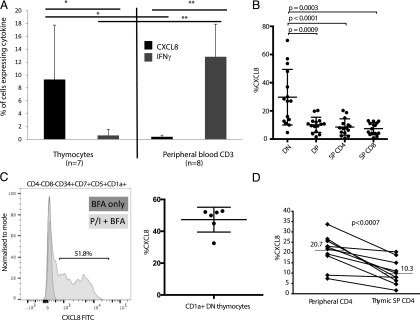
CXCL8 production is imprinted in the thymus. CXCL8 production was determined following activation in vitro with PI (4 h, in the presence of BFA) in whole thymocytes versus peripheral blood from humanized mice (37 wk post–hematopoietic stem cell inoculation, *n* = 7) (**A**), sorted human thymocyte subsets (*n* = 15, CD4^−^CD8^−^ DN, CD4^+^CD8^+^ double positive, CD4^−^CD8^+^ SP CD8, CD4^+^CD8^−^ SP CD4^+^) (**B**), and enriched T-lineage–committed DN thymocytes (defined as CD4^−^CD8^−^CD34^+^CD7^+^CD5^+^CD1a^+^, *n* = 6) (**C**). Example and cumulative data are shown. Stimulation: BFA only in dark gray and PI+BFA in light gray. (**D**) Comparison of CXCL8 production between paired human SP CD4^+^ thymocytes and naive (CD31^+^CD45RA^+^) peripheral CD4^+^ T cells (*n* = 10). *p < 0.05, **p < 0.001.

Given that CXCL8-producing CD4^+^ T cells are enriched among RTEs, it was appropriate to ask whether CXCL8 expression was induced during T cell development. Indeed, following PI-induced activation in vitro, CXCL8 was expressed by ∼10% of total thymocytes harvested from humanized mice at 37 wk post–hematopoietic stem cell inoculation, a time point at which CXCL8 expression in the periphery of mice was greatly diminished (Fig. 2A).

To determine at which stage(s) in human T cell development CXCL8 expression became evident, thymocyte subsets were sorted from pediatric human thymus and activated with PI in vitro. Cells expressing neither CD4^+^ nor CD8^+^ (double-negative [DN] cells) contained the most immature T cell precursors and were most enriched in CXCL8 producers (mean 29.7%, *n* = 15) (Fig. 2B). Thereafter, CXCL8 producers declined (Fig. 2B).

Because the thymocyte DN subset is highly heterogeneous, it was re-examined, using coexpression of CD34, CD5, CD7, and CD1a to identify T-committed cells. An average of ∼50% of such cells were CXCL8 producers (*n* = 6 thymuses) (Fig. 2C). Given that CXCL8 is produced by myeloid cells, it was not surprising that CXCL8 production was also observed in CD1a^−^ DN thymocytes that retain myeloid lineage potential (Supplemental Fig. 1). Next, CXCL8 production was examined in CD4^+^ single-positive (SP) thymocytes versus paired naive peripheral CD4^+^ T cells containing RTEs. Interestingly, the latter harbored the greater percentage of CXCL8 producers (*p* < 0.0007) (Fig. 2D), strongly suggesting that SP thymocytes that exit the thymus are enriched in those expressing CXCL8.

### CXCL8-producing CD4^+^ T cells are distinct

**FIGURE 3.**
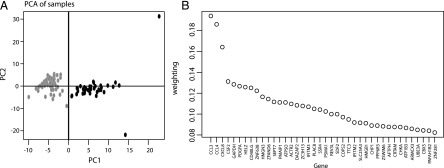
Results of single-cell RNA sequencing. (**A**) PC analysis of expression data. Each point represents a sample whose expression data has been projected onto the first two PCs for the expression data subset that includes all genes with statistically significant differential expression. (**B**) Genes providing the largest contribution to weightings for PC1.

To better understand CXCL8-producing T cells, CD4^+^ cells passively purified from CB by negative selection were activated with PI, and single cells were captured using the C1 Single-Cell Auto Prep System (Fluidigm). A total of 96 single cells (55 from one CB and 41 from another) were selected for full transcriptomic analysis. Total gene counts were normalized to ERCC spike-in control counts to assess cellular RNA content and sequencing depth. Unbiased investigation of the data by principal component (PC) analysis identified two distinct clusters of cells (gray and black, Fig. 3A). Of note, CXCL8 expression was one of the strongest contributors to PC1 (*x*-axis in Fig. 3A), thereby driving the discrimination of the two cell types. Contributing to this discrimination were RNAs encoding two chemokines, CCL3 and CCL4 (macrophage inflammatory protein-1α and -1β), which are expressed by CD4^+^ Th1 cell progenitors in mice (Fig. 3B, see *Discussion*). Spearman rank correlation (Table I) showed that CXCL8 expression was most strongly associated with the T-bet–inducing transcription factor *EGR1* (Spearman rank correlation = 0.55), *PDGFA* (Spearman rank correlation = 0.54), and *GMCSF* (*CSF2*; Spearman rank correlation = 0.49). Approximately one fifth of GM-CSF–producing T cells in infants coproduced CXCL8, whereas ∼3% of CXCL8-producers coproduced GM-CSF (Supplemental Fig. 2), consistent with infants harboring more CXCL8-producing cells than GM-CSF–producing cells. Indeed, the situation was reversed in adults (Supplemental Fig. 2), consistent with GM-CSF producers now outnumbering CXCL8-producing cells.

**Table I.** Spearman rank correlation: top 50 gene associations with CXCL8 expression

Rank	Gene A	Gene B	Correlation
1	CXCL8	EGR1	0.5549
2	CXCL8	PDGFA	0.53694
3	CXCL8	CCL3	0.50176
4	CXCL8	CSF2	0.49463
5	CXCL8	AFTPH	0.48789
6	CXCL8	EGR3	0.48369
7	CXCL8	GAPDH	0.47071
8	CXCL8	CCL4	0.46395
9	CXCL8	SDF2	0.43786
10	CXCL8	BTLA	−0.4369
11	CXCL8	CHKA	0.42098
12	CXCL8	ATP5O	0.42012
13	CXCL8	HELZ	0.41729
14	CXCL8	ZNF638	0.41004
15	CXCL8	AKAP13	0.39971
16	CXCL8	PLP2	0.39489
17	CXCL8	PLAC8	0.39369
18	CXCL8	PIKFYVE	0.39082
19	CXCL8	AP3D1	0.38813
20	CXCL8	RPS12	0.38720
21	CXCL8	DAZAP2	0.38410
22	CXCL8	PLAU	0.38267
23	CXCL8	ZC3H13	0.37866
24	CXCL8	SSR4	0.37593
25	CXCL8	ARID5B	−0.3758
26	CXCL8	DUSP10	0.37505
27	CXCL8	PSMA1	0.37081
28	CXCL8	ZSWIM6	0.37014
29	CXCL8	IFITM3	0.36902
30	CXCL8	AGO2	−0.36722
31	CXCL8	FAM46A	0.36690
32	CXCL8	CALM1	−0.36385
33	CXCL8	TMF1	0.36253
34	CXCL8	CAP1	0.36166
35	CXCL8	MTRNR2L8	0.36091
36	CXCL8	TXNL1	0.35475
37	CXCL8	NFKBIA	0.35448
38	CXCL8	HMGN3	0.35418
39	CXCL8	CARD8	0.34913
40	CXCL8	AKIRIN2	0.34817
41	CXCL8	CRTAM	0.34734
42	CXCL8	SREK1IP1	0.34725
43	CXCL8	CD40LG	−0.34713
44	CXCL8	BMPR2	0.34449
45	CXCL8	MTRNR2L2	0.33626
46	CXCL8	ACAP2	0.33592
47	CXCL8	ATP5A1	0.33311
48	CXCL8	IFITM2	0.33230
49	CXCL8	RAN	−0.3313
50	CXCL8	SLC25A3	0.3279

### CXCL8^+^CD4^+^ T cell conversion to IFN-γ producers

To better understand the fate of CXCL8-producing T cells, we tracked the effector functions of bulk-activated, sorted, naive CB CD4^+^ T cells ex vivo. Although such cells produced only CXCL8 upon initial stimulation with PI ex vivo, prolonged stimulation for 5 d with anti-CD3 plus anti-CD28 led to the appearance of IFN-γ, particularly among cells that had divided, as reflected by CellTrace Violet dilution (Supplemental Fig. 3A). When cells were stimulated with IL-7 alone, a critical factor for naive T cell survival, there was less proliferation, but an even greater percentage of divided cells expressed IFN-γ (Supplemental Fig. 3A). By gating on discrete generations from several CB CD4^+^ T cell samples (*n* = 4/5), it was evident by the third cell division that the percentage of IFN-γ−expressing cells was significantly different from the percentage of IFN-γ−producing cells among undivided CD4^+^ T cells (Supplemental Fig. 3A). Furthermore, following several rounds of proliferation, T-bet was induced and was largely attributable to those CD4^+^ cells expressing IFN-γ (Supplemental Fig. 3B). Similarly, the decline over time of cells expressing CXCL8 in humanized mice (Fig. 1C) was reciprocated by an increased percentage of cells producing IFN-γ (Supplemental Fig. 4).

**FIGURE 4.**
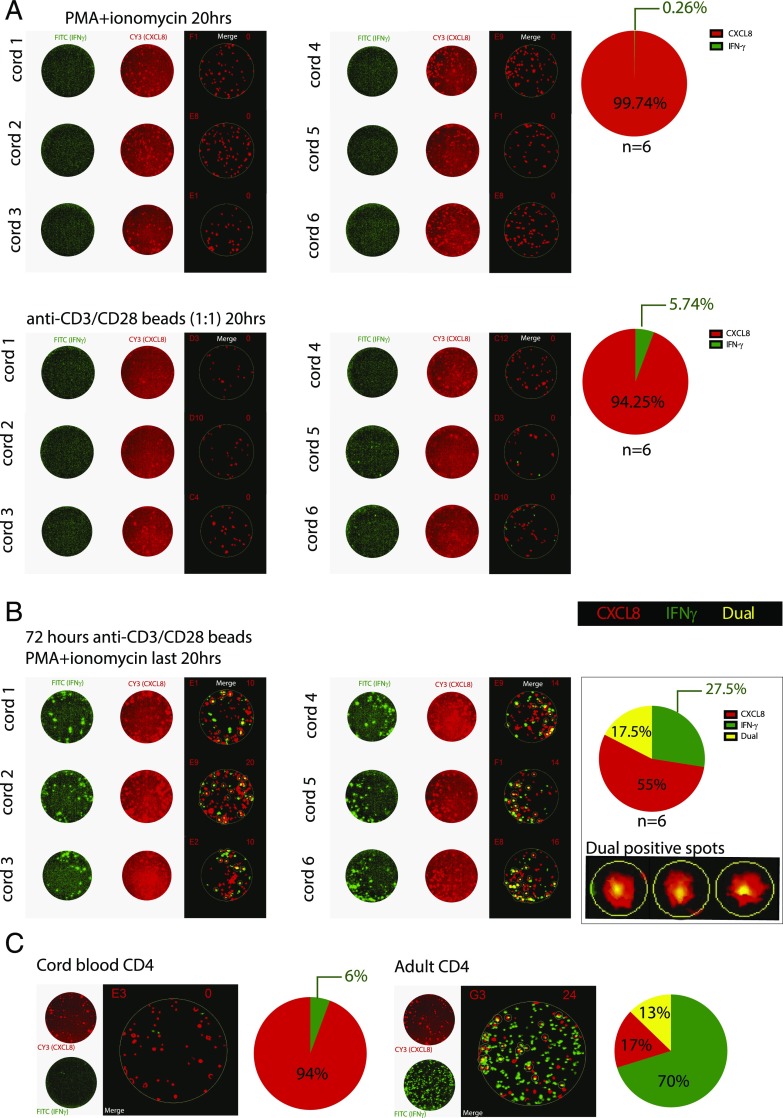
CXCL8^+^ CD4^+^ T cells are precursors for conventional Th1 effectors. CXCL8/IFN-γ production on a per-cell basis is shown by the FluoroSpot assay in which green spots represent IFN-γ production, and red spots represent CXCL8 production. A merged image to show dual-positive spots is shown for each case; the number of dual-positive spots in the well is annotated on the top right of each image. Corresponding pie charts depict the percentage of IFN-γ^+^ (green), CXCL8^+^ (red), and CXCL8^+^IFN-γ^+^ (yellow) spots from all of the samples. Naive CD4^+^ T cells isolated from six cord samples were stimulated ex vivo (20 h PI or anti-CD3+CD28 beads) (**A**) and for 72 h (anti-CD3+28 beads, followed by PI restimulation for the last 20 h) (**B**). All experiments were performed in duplicate at a range of cell titrations. All wells depicted had between 7.5 and 22.5 × 10^3^ cells per well. Examples of dual-positive spots are shown at a higher magnification (right panel). (**C**) Overnight PI stimulation (10,000 sorted CD4^+^ T cells per well) for cord and for adult CD4^+^ T cells. Corresponding pie charts depict the percentage of IFN-γ+ (green), CXCL8+ (red), and CXCL8+IFN-γ+ (dual-yellow) spots.

To determine whether this phenotypic transition might be due to a direct conversion of CXCL8-producing cells into those producing IFN-γ, a dual-ELISPOT (IFN-γ/CXCL8 FluoroSpot) system was adopted, in which different cytokines produced by individual cells over the time-course of the assay are captured in situ on a polyvinylidene difluoride membrane. PI activation of naive CD4^+^ T cells sorted from several CB samples (*n* = 8) primarily induced CXCL8 (99.74%) over an initial 20-h period (Fig. 4A). Use of anti-CD3+CD28 beads to activate sorted naive CD4^+^ T cells induced slightly more (<6%) IFN-γ–producing cells (lower panel, Fig. 4A), but neither PI nor anti-CD3+CD28 induced any dual-positive cells (i.e., there was a lack of yellow spots) among CB cells. However, when naive CD4^+^ T cells were allowed to proliferate by sustained activation for 52 h with anti-CD3+CD28, prior to PI stimulation, the percentage of IFN-γ^+^ spots was increased, and more than one third were CXCL8^+^IFN-γ^+^ dual-positive cells (yellow spots, Fig. 4B). The fact that no dual-positive cells were detected at 20 h argues against the assay being confounded by the supposition of two cells with different effector functions. Rather, it strongly suggests that some single cells acquired a second effector function over time.

As would be expected, PI activation of adult (age = 24 y) CD4^+^ T cells primarily provoked IFN-γ production (green spots; Fig. 4C, right panel) in contrast to CXCL8 production by CB-derived CD4^+^ T cells (red spots; Fig. 4C, left panel), However, adult CD4^+^ T cells included some CXCL8 producers (red spots; Fig. 4C, right panel), of which almost 50% were also IFN-γ^+^ (yellow spots; Fig. 4C, right panel). Taken together, the data suggest that the capacity for CD4^+^ T cells to coproduce IFN-γ and CXCL8 exists in adults and in neonates, raising the question of whether this reflects a developmental transition from CXCL8 production to IFN-γ production.

**FIGURE 5.**
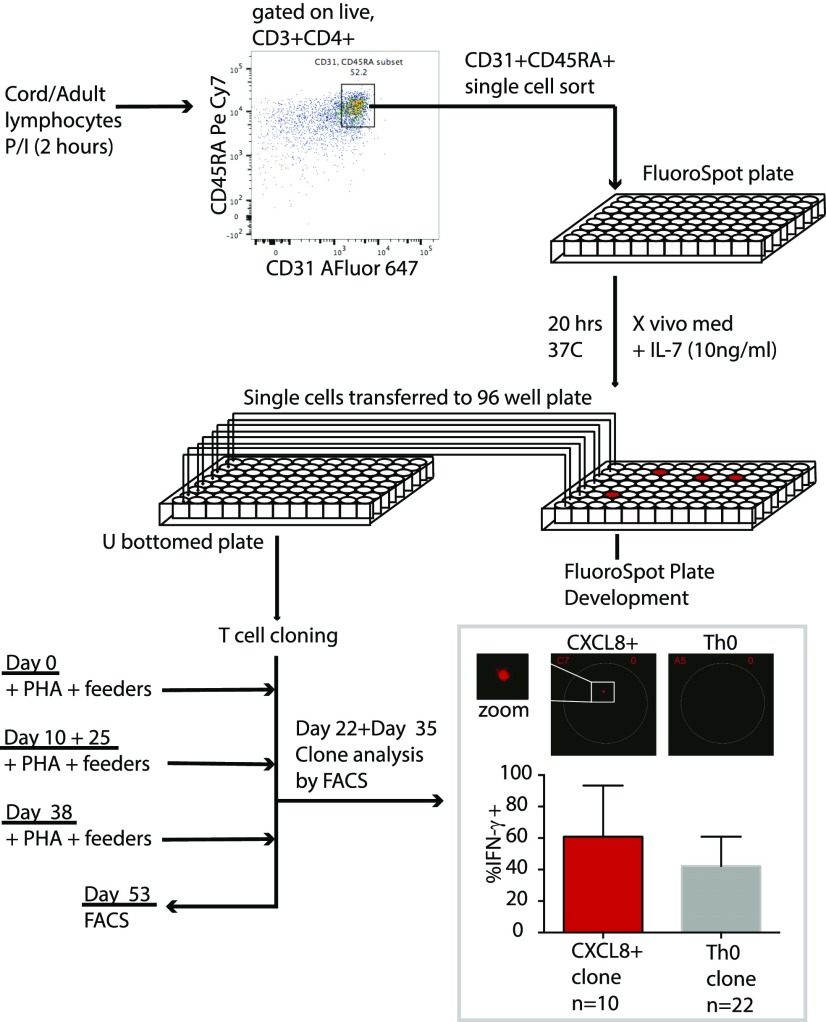
Schematic diagram demonstrating how single-cell sorting in FluoroSpot plates was performed. Inset: production of IFN-γ is shown for 10 clones derived from CXCL8^+^ cells and 22 clones derived from Th0 cells, with a representative FluoroSpot well shown above for a CXCL8^+^ well and a Th0 well. Clones were harvested after sufficient cell numbers had been reached and were stimulated with PI for 4 h prior to staining and acquisition.

To test this, it would be appropriate to clone single CXCL8^+^ cells and track them. However, no capture system for CXCL8-secreting cells exists. Therefore, activated naive CD4^+^ T cells were sorted as single cells directly into 96-well FluoroSpot plates, in which system a single red spot in a FluoroSpot well acts as a read-out for a CXCL8 producer. However, rather than discarding the individual cells prior to plate development, they were transferred to the corresponding well of a 96-well U-bottom plate and then cloned via several rounds of stimulation with PHA and irradiated feeder cells (Fig. 5). This allowed the fates of single CXCL8-producing cells to be tracked.

**FIGURE 6.**
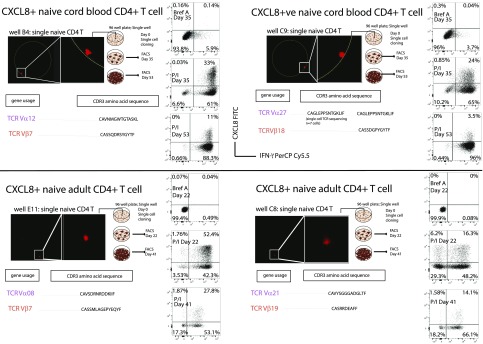
Clones derived from a single CXCL8-producing cell produce IFN-γ. Activated (2 h PI) naive CD4^+^ T cells were single-cell sorted directly into 96-well FluoroSpot plates. Single cells were recovered from the FluoroSpot plate and added to PHA (4 μg/ml) and irradiated feeder cells for cloning. The FluoroSpot plate was developed to reveal a signal left by a single CXCL8-producing cell. Cells cloned were subsequently stimulated (PI; 4 h) at two time points, and dot plots depicting IFN-γ (*x-*axis) and CXCL8 (*y*-axis) production at each time point are shown. To show clonality, sequencing of TCRα- and β-chains was performed using DNA extracted from sorted live 7-aminoactinomycin D^−^CD3^+^CD4^+^ T cells. The amino acid sequence for the CDR3 region and gene usage are shown for each clone.

**FIGURE 7.**
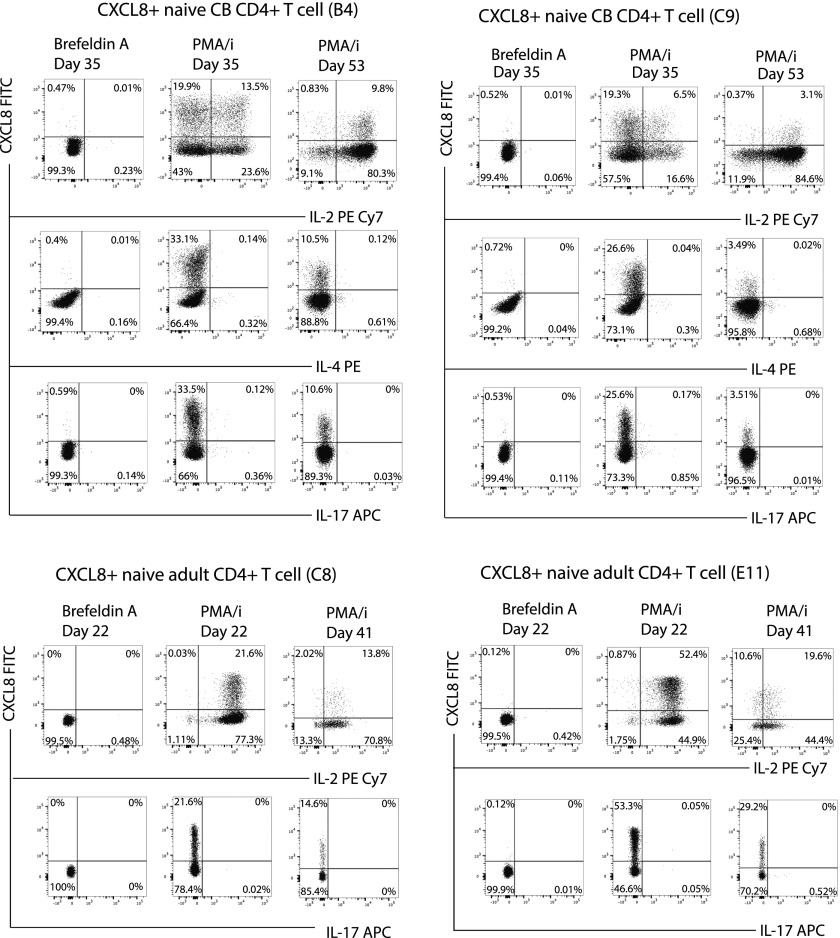
Effector function of clones derived from single CXCL8-producing T cells. Naive CD4^+^ T cells were single cell sorted into FluoroSpot plates for 20 h (2 h preincubation with PI), following by transfer to 96-well plates and cloning by addition of irradiated feeder cells and PHA (4 μg/ml). Once the FluoroSpot plate was developed, it was possible to determine which wells had only a single CXCL8^+^ spot. Subsequent effector function (IL-4/IL-2/IL-17A) in clones from these CXCL8-producing cells was then tracked over two time points by FACS. Two CB clones and two adult clones are shown.

Clones derived from 10 independent CXCL8-producing cells, isolated from adults and from CB, were expanded in this way, and investigated for cytokine expression following PI stimulation once sufficient cell numbers had been reached (Figs. 5–7). In parallel, the capacity to produce IFN-γ was scrutinized for 22 clones derived from wells in which neither CXCL8 nor IFN-γ was initially produced (i.e., analogous to a Th0 phenotype). All 10 clones derived from CXCL8-producing cells went on to produce IFN-γ. In so much as it could be assessed, IFN-γ–producing cells were obtained with comparable efficiencies from CXCL8^+^ clones and Th0-derived clones, and the cloned lines derived in each case showed comparable levels of IFN-γ production (Fig. 5).

Certain clones (those that were being expanded further to derive sufficient cell numbers for TCR sequencing) were followed for a longer period, through to days 35 and 53. At both time points, a substantial fraction (often ∼90% of cells) of each clone produced IFN-γ (Fig. 6) and many also produced IL-2 (Fig. 7). Of note, the cultures coproduced CXCL8, but this declined over time (Fig. 6), consistent with the time-dependent appearance of IFN-γ single-producers in the dual-ELISPOT assay (above) and with the reduction in CXCL8 production with age (Fig. 1). Thus, the mean ratio of IFN-γ^+^ (single-producers) to CXCL8^+^IFN-γ^+^ (double producers) prior to day 38 (addition of fourth round of feeders) was 2:1 (*n* = 5 clones), whereas this increased at later time points to a mean of 6:1 (*n* = 4). In contrast, there was no reproducible evidence for single CXCL8-producing cells giving rise to IL-17A or IL-4 producers (Fig. 7), although under the standardized cloning conditions used, we and other investigators have observed the generation of T cell clones with a Th2 phenotype.

Having established that cells derived from a single CXCL8 producer could express IFN-γ alone or be dual IFN-γ/CXCL8 producers, it was important to verify the cells’ clonality. Adult clones E11 and C8 were sampled for DNA at day 41, and CB clones C9 and B4 were sampled at day 53, whereupon sequencing identified, in every case, a single α- and β-chain (Vα27-Vβ18 [C9], Vα12Vβ7 [B4], Vα8Vβ7 [E11], and Vα21Vβ19 [C8]), with single CDR3 sequences for each chain (Fig. 6). Moreover, the TCRα-CDR3 sequence for C9 was identical to that identified when seven single cells of clone C9 were sequenced on day 38. These data confirm that each culture represents a clonal population derived from single CXCL8-producing CD4^+^ T cells that progresses to include IFN-γ–producing cells and CXCL8/IFN-γ dual producers. Thus, Th1 effector cells can derive from single CXCL8-producing CD4^+^ T cells that are common among RTEs of babies, children, and adults.

## Discussion

The data presented in this study show that the primary effector potential enriched in CD4^+^ RTEs in adults, as well as in children, is CXCL8 production and that such T cells may convert to a classic adaptive Th1-type effector cell following sustained proliferation, as would occur following Ag priming in the lymph nodes. Thus, these data highlight that CXCL8 production does not represent end-stage differentiation; instead, CXCL8^+^ cells can differentiate into a more conventional T cell phenotype. This model of human T cell differentiation is in stark contrast to the current model by which “functionless” Th0 cells are the progenitors of Th1 cells. Nonetheless, several data sets are consistent with our findings.

Most directly, bulk human CXCL8-producing cells were shown to give rise to IFN-γ–producing cells following TCR activation and/or cytokine-induced proliferation in vitro; however, because the analysis was not undertaken at a single-cell level, bona fide phenotypic conversion could not be distinguished from the outgrowth of existing IFN-γ–producing cells (23, 24). In a parallel study, it was shown that mouse Th1 cells could derive from T cells producing IL-17 (7). Interestingly, mouse IL-17–producing cells may, in several ways, be parallel to human CXCL8-producing T cells, including their capacity to activate neutrophils, a major function of human CXCL8 that is largely assumed by IL-17 in mice (which lack the CXCL8 gene) (8, 9). Third, *CCL3* and *CCL4* gene expression cosegregate with *CXCL8* expression, just as they are markers of CD4^+^ Th1 progenitors in mice (25).

The capacity of a T cell to deliver effector function prior to clonal proliferation and Th1 differentiation would seem a highly efficient device to provide a rapid innate-like barrier to bacterial dissemination that would be particularly useful in neonates in whom RTEs are preferentially enriched. This inner core of innate activity is shed in favor of an adaptive Th1 end state for cells provoked into sustained proliferation by Ag stimulation and coreceptor signaling, which simultaneously provide the necessary quality control to limit autoimmunity. The strong correlation of *CXCL8* gene expression with *EGR1* gene expression highlights the propensity for Th1 skewing of CXCL8-producing cells because EGR1 is a regulator of *Tbx21* gene (T-bet) induction during Th1 polarization (26). Indeed, human T-BET protein induction could be observed in CB-derived CD4^+^ cells after several rounds of proliferation, coincident with the competence to express IFN-γ. Whether the conversion to IFN-γ production is T-BET dependent in these cells has not been formally assessed. Possibly the switch to aerobic glycolysis induced by sustained proliferation is a contributory driver of conversion (27). In so much as it could be assessed in this study, clones derived from CXCL8^+^ CD4^+^ T cells seemed comparable to their Th0 counterparts in generating Th1 effectors. Likewise, although we provide clear evidence that single CXCL8^+^ CD4^+^ T cells can give rise to IFN-γ–producing CD4^+^ T cells, we do not exclude their potential to also differentiate into more diverse effector T cell types.

Our demonstration, by TREC analysis, that CXCL8 production is a feature of RTEs is also consistent with the observation of diminished CXCL8 production in thymectomized children (24). However, the data might be viewed as inconsistent with the proposed discontiguity of fetal/neonatal and adult immune systems, with regulatory T cell (Treg) generation considered a default pathway for neonatal T cells (5, 28). Indeed, very few genes scoring significantly in our single-cell analysis of CB T cells were related to Treg function. However, the data sets might be reconciled if Treg function, like IFN-γ function, arises primarily after cell proliferation, thereby to limit the potency of emerging effector T cells. Indeed, in almost all systems in which they have been examined, Treg frequencies correlate with those of CD4^+^ T effector/memory cells (29, 30).

Although CXCL8 expression was acquired in the thymus by T-lineage–committed DN cells, prior to TCR signaling, a larger percentage of CD4^+^ RTEs expressed CXCL8 compared with their paired SP CD4^+^ thymocyte counterparts. CXCL8 production might be enhanced in RTEs by peripheral factors that are dependent on the milieu present during primary Ag recognition (31, 32). Alternatively, because only a small subset of SP CD4^+^ thymocytes are capable of proliferation, thymic egress, and cytokine production (33), CXCL8 expression may correlate with factor(s) that mark the competence of cells to exit the thymus (24); indeed, CXCL8 might be a surrogate marker for such competence. In agreement with this, we have observed a 2–3-fold enrichment in CXCL8-producing cells within thymic CD4^+^ T cells that express S1PR1 compared with S1PR1^−^ cells (A. Das and D.L. Gibbons, unpublished observations).

Finally, it is noteworthy that T-ALL of different stages also produced CXCL8, given that CXCL8 has been established as an indicator of poor prognosis (34). These findings might indicate that expansion of “immature” CXCL8-producing T cells, coupled with their failure to differentiate toward Th1 cells, is a pathognomonic feature of the disease, meriting further investigation.

## Supplementary Material

Data Supplement
