# Supplementary material for: Adaptive from Innate: Human IFN-γ+CD4+ T Cells Can Arise Directly from CXCL8-Producing Recent Thymic Emigrants in Babies and Adults
Source: J Immunol. 2017 Jul 28;199(5):1696–705. doi: 10.4049/jimmunol.1700551 (PMC5563168; doi:10.4049/jimmunol.1700551)
Supplement: Data Supplement [file JI_1700551.zip › JI_1700551_Supplemental_Figures_1.pdf]

# Das et al, Supplementary material JI 2017

**Supplementary Figure 1**

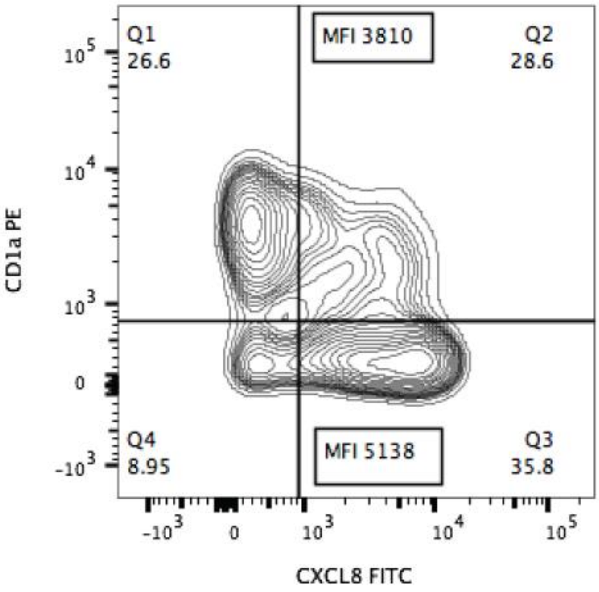


**Supplementary Figure 1. CXCL8 is produced by both CD1a negative and CD1a positive DN thymocytes.**

CD1a is a marker of T cell lineage commitment. Shown here is a representative FACS plot, demonstrating CXCL8 production in DN thymocytes following ex vivo stimulation with PI against CD1a expression. CXCL8 production (shown as a percentage and mean fluorescence intensity MFI) within the CD34+CD7+CD5+CD1a negative population is consistent with the myeloid lineage potential of these cells.

**Supplementary Figure 2**

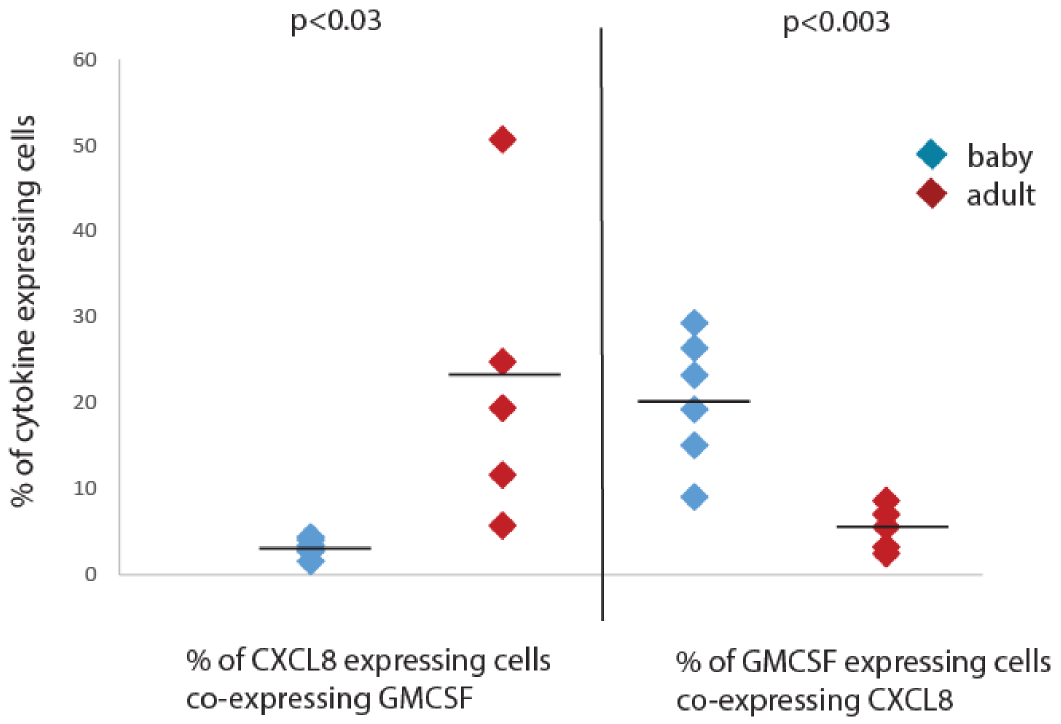


**Supplementary Figure 2**

Peripheral blood cells isolated from preterm infants (blue diamonds) or adults (red diamonds) were activated with PI (in presence of BFA) for 4hrs and then stained for surface expression of TCRαβ and CD4 and intracellular expression of GMCSF and CXCL8. Results represent % of GMCSF expressing CD4 T cells that co-express CXCL8 and *vice versa* from the different starting cell populations.

**Supplementary Figure 3**

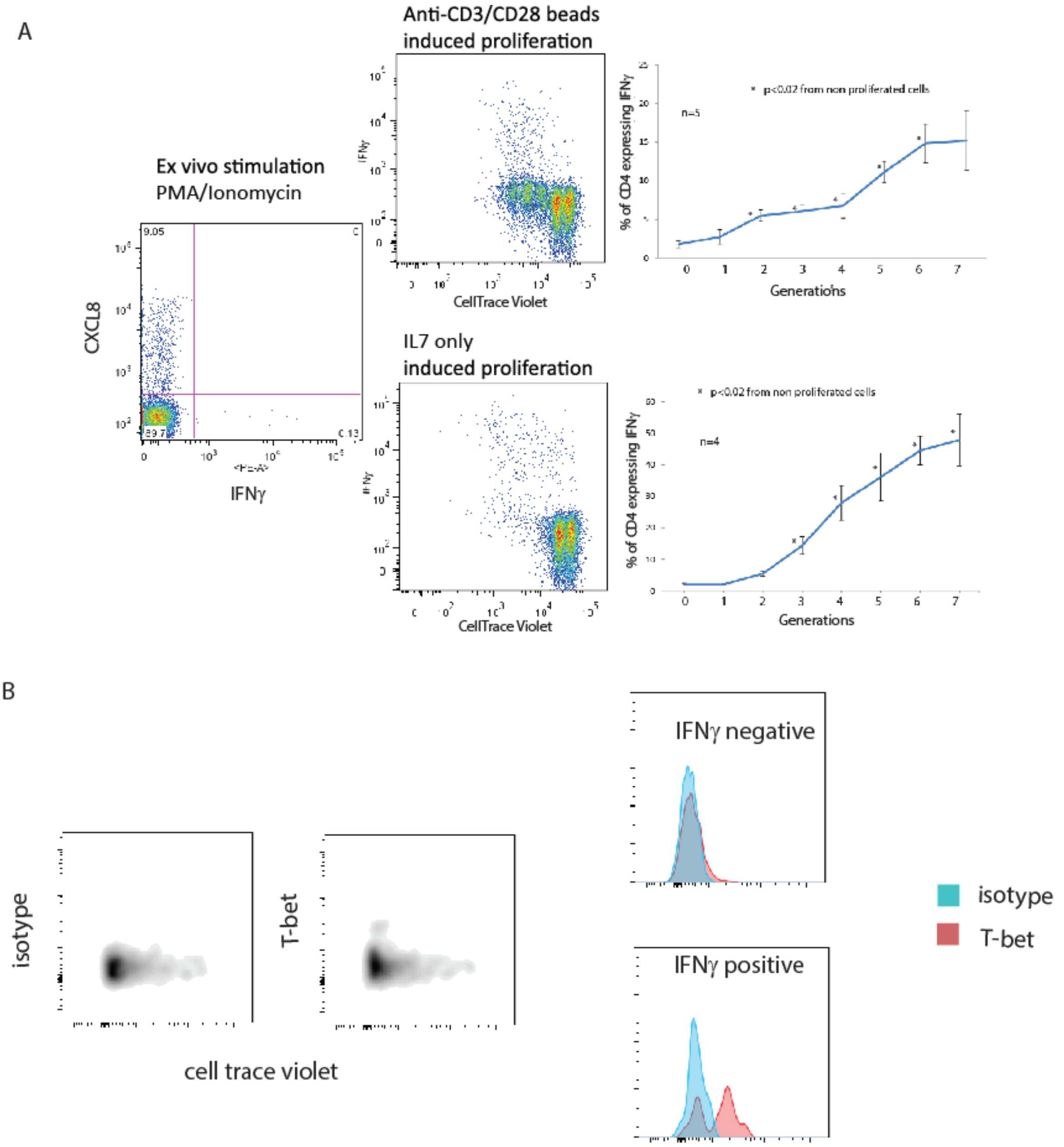


**Supplementary Figure 3. Tracking the fate of neonatal CXCL8$^+$ CD4$^+$ T cells**

A. Stimulation *ex vivo* with PI induced CXCL8 but not IFN-γ (representative FACS plot shown). To track the fate of these cells, cord CD4+ T cells were labeled with cell trace violet (x axis) and left for 5 days with anti-CD3+CD28 beads or with IL7 alone to induce proliferation followed by restimulation with PI. Graphs to the right depict the mean % of IFN-γ production in each successive generation following PI re-stimulation.

B. Density plots show total CD4 T cells stained with isotype (left) or T-bet (right) 5 days post labeling with cell trace violet and stimulation with anti-CD3+CD28 beads. Following re-stimulation with PI, T-bet expression was analysed in IFN- γ negative (top histogram) or IFN- γ positive (bottom) CD4 T cells

**Supplementary Figure 4**

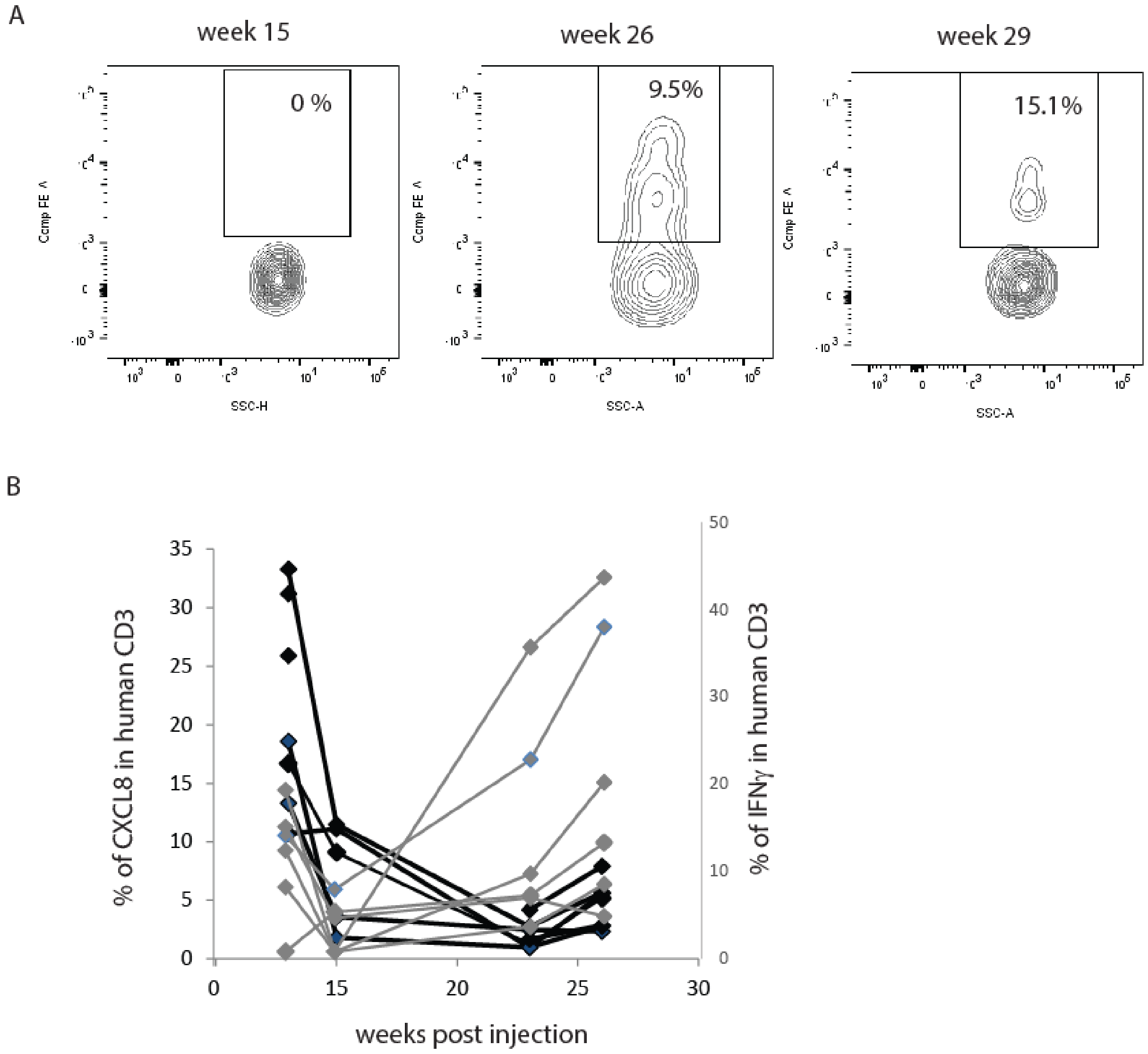


**Supplementary Figure 4. IFN-γ expressing T cells increase in humanised mice over time.**

Irradiated NSG mice were reconstituted with human CD34+ cells (cord blood derived).

A. FACS plots show an example of IFN-γ production within $CD3^+$ T cells (PI + BFA for 4 hrs) in one reconstituted NSG mouse over time (the same mouse as shown in Fig 1c);
B. The graph shows decline in T cell production of CXCL8 (black lines) over time in individual mice with a reciprocal increase in T cell production of IFN- γ (grey lines)
